# Evaluation of Usefulness of hs-CRP and Ferritin Assays in Patients with Nasal Polyps

**DOI:** 10.1155/2014/794060

**Published:** 2014-03-11

**Authors:** Robert Partyka, Jacek Pałac, Zbigniew Paluch, Bożena Szyguła-Jurkiewicz, Grzegorz Namysłowski, Maciej Misiołek, Przemysław Jałowiecki, Danuta Kokocińska

**Affiliations:** ^1^Clinical Division of Anesthesiology and Intensive Treatment of the Department of Anesthesiology, Intensive Treatment and Emergency Medicine, Medical University of Silesia in Katowice, Plac Medyków 1, 41-200 Sosnowiec, Poland; ^2^ENT Department in Zabrze, Medical University of Silesia, Katowice, Poland; ^3^3rd Department and Clinical Division of Cardiology SUM, Katowice, Poland

## Abstract

*Background.* Chronic nature of the nasal polyps, tendency to recurrence, and lack of satisfying treatment need the diagnostic's parameters which show early inflammatory state as ferritin and hs-CRP. *The Aim of Study.* Assessment of hs-CRP and ferritin blood levels in nasal polyps patients in evaluation of treatment efficacy. * Methods.* All 38 patients were divided into 2 groups. Group I included 19 patients with anti-inflammatory therapy 2 weeks after surgery. Group II included 19 patients without anti-inflammatory therapy 2 weeks after surgery. The levels of hs-CRP and ferritin have been assessed before and 2 and 6 weeks after surgical treatment. *Results.* Research showed statistically significant difference of ferritin's concentration between examined groups 6 weeks after surgery (*P* < 0.05) and statistically significant difference of hs-CRP concentration 2 and 6 weeks after surgery (*P* < 0.05). *Conclusion.* (1) The analysis of serum ferritin and hs-CRP concentrations can be useful in early postoperative detection of inflammatory state in patients with nasal polyps and for the effectiveness of therapy. (2) Lack of correlation between mean ferritin and hs-CRP serum levels, at each diagnostic and monitoring stage, shows that they are independent and cannot be determined interchangeably.

## 1. Background

The chronic character of the disease, the distressing symptoms, the recurrent polypoid lesions, and the absence of satisfactory treatment methods all make chronic paranasal sinusitis with nasal polyps a serious social, clinical, and economic problem. Epidemiological studies show that more than a third of the population suffers from hypertrophic polypoid nasal and sinonasal mucosa; in some patients, the lesions have no clinical manifestations, whilst in one case in fifteen, progression to the full disease occurs. The annual incidence of the full disease is 0.63 cases per 1,000. It occurs more frequently in men (more than 2-fold), with peak incidence in their fifties, that is, in 1–4% of the population [1]. Despite advances in molecular biology, the pathogenesis of polyps still remains unclear. Epithelial cell dysfunction and eosinophilic inflammation of the upper respiratory tract mucosa are thought to play a crucial role in the development of nasal polyps [2, 3]. Attention has also been drawn to the impact of bacteria and fungi in the creation of polypoid nasal and sinonasal lesions [3, 4].

Nasal polyps are treated with surgery (functional endoscopic nasal surgery) and with anti-inflammatory pharmacotherapy, or with a combination of the two. The choice of pharmacotherapy is determined by the cytological type of the polyp: in the case of eosinophilic polyps, the best treatment results are obtained with local and systemic glucocorticosteroid therapy [5–7].

### 1.1. Acute-Phase Proteins

A review of the available literature shows that researchers have paid a great deal of attention to inflammatory response markers. Cytokines, such as IL-1 and TNF-*α*, and acute-phase proteins are assessed to predict treatment outcome. It has not diagnosed yet the suitability of determinations of hs-CRP and ferritin in patients with nasal polyps.

### 1.2. C-Reactive Protein

CRP is not only an inflammatory marker but also an important factor that affects the development of inflammation [8–10]. High serum levels of CRP are characteristic of an acute inflammatory condition; CRP measurement with a high sensitivity CRP (hs-CRP) between 0.5 and 10.00 mg/L facilitates the detection of low-intensity chronic inflammatory processes.

### 1.3. Ferritin

Ferritin is a water-soluble protein that stores iron; it is composed of a spherical protein shell called an apoferritin and a mineral core [11]. The assessment of ferritin levels is important not only in classic diseases related to iron uptake, transport, and storage, but also in diseases characterized by inflammatory and infectious processes and tissue damage and repair [12].

## 2. Aim of Study

The aim of this work is to assess the usefulness of serum assays of hs-CRP and ferritin in predicting the efficacy of treatment in patients with nasal polyps.

## 3. Material and Methods

The study encompassed 38 adults (men and women) who were undergoing treatment for chronic paranasal sinusitis for the first time. The patients were divided in two groups.

Group I consisted of 19 patients, 4 women and 15 men (mean age 44.2 ± 12.8), who were treated surgically and were receiving anti-inflammatory treatment two weeks after the surgery.

Group II consisted of 19 patients, 7 women and 12 men (mean age 49.9 ± 13.8), who were treated only surgically, in some cases receiving anti-inflammatory treatment after six weeks from the surgery and after the determination of the markers being studied.

Patients of groups I and II qualified for the study were in a similar state of clinical advancement, no postoperative complications and additional inflammatory.

Excluded from the study were patients with diagnosed (1) acute and chronic inflammatory states of an infectious etiology, with the exception of an inflammatory state of the upper respiratory tract, (2) diseases of autoaggression, (3) tumors, and (4) recurrent nasal polyps, as well as individuals who were receiving iron preparations.

Serum hs-CRP and ferritin were assayed in all patients. Fasting blood samples were taken from the median basilic vein. After centrifugation, blood serum for the assay was stored at −20°C. The assays were carried out before treatment and then two and six weeks from the beginning of treatment. hs-CRP was assayed by chemiluminescence with a constant phase, using the commercially available kit by Siemens and an Immulite 1000 apparatus (Germany). A concentration of ≤3 mg/L was considered normal for healthy individuals.

Serum ferritin levels were assayed with the MEIA method, using the commercially available kits by Abbott and an Architect i2000 apparatus (USA). A concentration between 45 and 140 ng/mL was considered normal for healthy individuals. The obtained results underwent statistical analysis; the level of statistical significance was *P* < 0.05.

## 4. Results

The patients qualified for the study were comparable with regard to age and gender.

Due to the normality of distribution of the obtained results, the Student's *t*-test was used. The obtained results and statistical significance are presented in Table 1.

**Figure 1:**
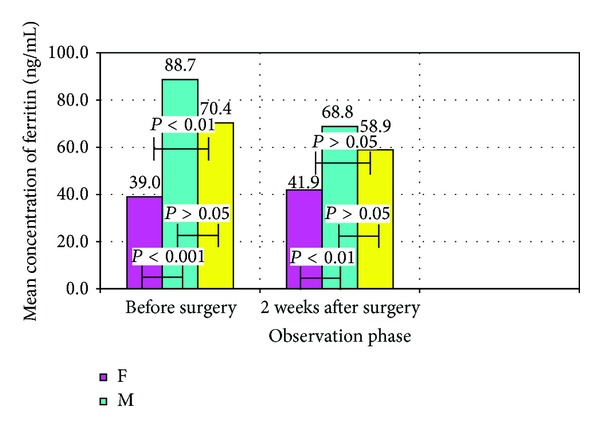
Mean concentrations of ferritin in women, men, and patients with total test results for two medium-sized.

Ferritin concentration was determined separately for men and women qualified for the study. The results are shown in Table 2 and in Figure 1. No statistical significance between serum ferritin men and women allows for a total assessment of ferritin in treatment groups I and II.

**Figure 2:**
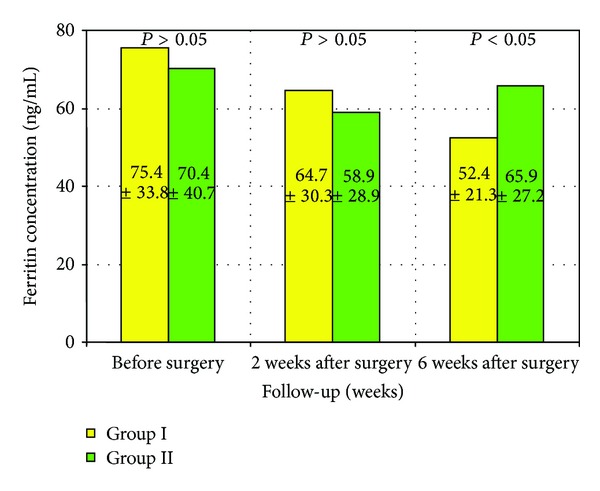
Ferritin concentrations in the groups studied over the analyzed follow-up period. Group I: patients treated with anti-inflammatory therapy 2 weeks after surgery. Group II: patients without anti-inflammatory therapy.

The mean values of ferritin concentrations in the groups studied over the analyzed follow-up period are presented in Figure 2.

There were no statistically significant differences in ferritin concentrations between the groups studied before surgery and two weeks after surgery. However, the difference was statistically significant at six weeks after surgery (*P* < 0.05). The concentrations of serum hs-CRP in the two groups studied are presented in Figure 2.

**Figure 3:**
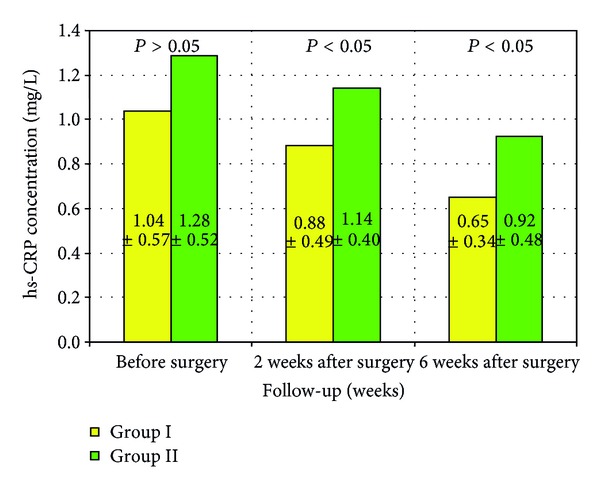
hs-CRP concentrations in the groups studied over the follow-up period. Group I: patients treated with anti-inflammatory therapy 2 weeks after surgery. Group II: patients without anti-inflammatory therapy.

The results of the assessment of mean hs-CRP concentrations differed from the analogous assessment carried out for ferritin. There were no statistically significant differences between the groups before surgery (*P* > 0.05), whilst at two and six weeks after surgery, the differences were statistically significant (*P* < 0.05) (Figure 3).

The last stage of the statistical analysis was an assessment of the correlation between the concentrations of ferritin and hs-CRP over the consecutive stages of the followup. The results of the analysis are presented in Table 3.

The correlation was not statistically significant at any stage of the followup, regardless of the treatment method used.

## 5. Discussion

Chronic paranasal sinusitis with nasal polyps is a serious social, clinical, and economic problem. The treatment involves surgery, pharmacotherapy, or a combination of the two. According to many authors, the best results are achieved with surgery, followed by pharmacotherapy. The high efficacy of the combined surgical and pharmacological anti-inflammatory treatment was confirmed by the study of Dijkstra et al. [13]. Still, the decision to use glucocorticosteroids requires modern and precise diagnostics, mainly with a view of monitoring the inflammatory process of the upper airway, thus monitoring the course of the disease.

Acute-phase proteins are systemic markers of inflammatory response that are used in the diagnosis and prognosis of the course of inflammatory processes.

The literature shows that acute-phase proteins are of interest to cardiologists, rheumatologists, neurologists, and nephrologists in the context of the etiopathogenesis and prognosis in diseases connected with chronic inflammatory processes. Ridker et al. have shown in 2003 that CRP, previously considered only in its inflammatory aspect, has become a strong and independent prognostic factor in the risk assessment of cardiovascular disease [14, 15].

Khreiss et al. in 2004 and Pasceri et al. have noted that CRP is required in diagnostic of proinflammatory effect in human endothelial cells [9, 10].

The literature also shows that another inflammatory marker that is currently of interest to many researchers is ferritin. F. M. Torti and S. V. Torti paid attention to the relation between ferritin and inflammatory infectious and neoplastic diseases with tissue damage or repair [12].

Millerot et al. have shown that high levels of ferritin correlate with very bad prognosis in patients with ischemic stroke [16]. In our study, we attempted to use ferritin and hs-CRP assays to monitor patients with chronic sinusitis and nasal polyps. The disease is characterized by the absence of a causal treatment and it is crucial to monitor and treat the inflammatory process that underlies the disease. Based on the information on the clinical importance of ferritin and hs-CRP assays, we endeavoured to evaluate the usefulness of the two inflammatory markers in patients with nasal polyposis.

The study encompassed 38 patients who were diagnosed with nasal polyps. The patients were divided into two groups of 19, which were comparable with regard to age and gender. Polyps were removed surgically in all patients. In both groups the serum concentrations of ferritin and hs-CRP were assayed before surgery and then at two and six weeks after surgery. Anti-inflammatory treatment started two weeks after surgery in group I and six weeks after surgery in group II.

The first parameter that was evaluated was ferritin. Its mean concentrations both before surgery and during the postoperative followup were within the wide range of normal values, that is, 45–140 ng/mL. The mean preoperative concentrations were 75.4 ng/mL in group I and 70.4 ng/mL in group II and were not statistically significant. The mean ferritin concentrations also did not differ significantly two weeks after surgery. Compared to the preoperative values, the mean ferritin concentrations were lower in both groups: 64.7 ng/mL in group I and 58.9 ng/mL in group II. The lowering of the mean concentrations was exclusively affected by the procedure of polyp removal.

In group I, glucocorticosteroid therapy was started two weeks after surgery and postoperative wound healing. Six weeks after surgery, a further decrease in ferritin concentration was observed only in patients in group I, who were receiving steroid therapy. In contrast, a slow increase in ferritin concentration was observed in group II, which may confirm the presence of an inflammatory process and may indicate the necessity of introducing prompt postoperative steroid therapy. Ferritin concentrations first reached the level of statistical significance after six weeks from surgery.

The results obtained from group II, which included patients with no anti-inflammatory therapy, were different. The mean ferritin concentrations were not significantly different throughout the entire followup. For many years, clinicians have claimed unanimously that anti-inflammatory therapy should be started as soon as possible after surgery. Yet, a literature review shows that no diagnostic parameter has been proposed to facilitate the evaluation of the efficacy of therapy or to estimate treatment duration.

The assessment of ferritin concentration shows that it can be used in postoperative monitoring of patients. An increase in serum levels means that anti-inflammatory treatment should be resumed in order to prevent the recurrence of nasal polyps. The fact that the ferritin concentration does not exceed normal values does not limit its use in monitoring patients. A constant increase within normal ranges suggests the presence of an inflammatory process and a need for anti-inflammatory therapy.

Despite the absence of reports on the usefulness of ferritin assays in patients with nasal polyps, for several years attention has been drawn to its prognostic value in ischemic diseases of the heart and the central nervous system with secondary reperfusion, atherosclerosis, neurodegenerative diseases, such as Alzheimer's disease and Parkinson's disease, an inflammatory state of the lungs, rheumatic diseases, preneoplasia, and malignant tumors [17–21]. In the Finnish study by Salonen, ferritin was found to be an independent risk factor for myocardial infarction [22]. Davalos et. al. [18] suggest that ferritin concentration assay may be useful in predicting the course of ischemic stroke, as well as in evaluating biochemical changes with an ischemic focus.

The other inflammatory marker that was studied was hs-CRP. As with ferritin, the concentration of this protein was determined in all patients before surgery and then at two and six weeks after surgery. The evaluation of hs-CRP was carried out by comparing the two groups studied and analyzing group I (with steroid therapy two weeks after surgery) and group II (no anti-inflammatory therapy) separately. The mean concentrations of hs-CRP were within normal ranges and before surgery were 1.04 mg/L in group I and 1.28 mg/L in group II and were not statistically significant.

Unlike ferritin, hs-CRP maintained the mean concentration after two weeks of followup. The mean concentrations in the two groups after two weeks were 0.88 and 1.14 mg/L, respectively, and after six weeks 0.65 and 0.92 mg/L, respectively, and were statistically significant (*P* < 0.05) at both stages of the followup.

It is difficult to state what hs-CRP concentration should be considered to be correct as the issue is still being discussed widely in leading scientific journals. The most likely cause of CRP concentrations between 2 and 10 mg/L is a subclinical inflammatory state.

The CRP concentration has been demonstrated to be useful in assessing both the severity of inflammation and treatment method in cases of acute upper and lower respiratory tract inflammation. In the point-of-care study by Cals et al. [23] concerning CRP and antibiotic prescription, a positive effect of CRP assays was shown. Wadhwa et al. have shown, in year 2013, prognostic determination value of hs-CRP and ferritin in young patients with acute myocardial infarction [24].

The assessment of the mean concentrations of ferritin and hs-CRP in the two groups studied here drew our attention to the difference in their profiles, and hence the correlation between ferritin and hs-CRP before surgery and during postoperative monitoring was assessed at two and six weeks after surgery. The obtained results are surprising. At no stage of the diagnosis and followup a correlation was observed between the inflammatory parameters being studied. The interpretation of this result is difficult as from a clinical point of view; both ferritin and hs-CRP are markers of an active inflammatory process. Presumably, their mechanisms differ.

Hence, further research is needed to continue the work started in this study and possibly to assess other inflammatory markers.

## 6. Conclusions

1. The analysis of serum ferritin and hs-CRP concentrations can be useful in early postoperative detection of inflammatory state in patients with nasal polyps and for the effectiveness of therapy.
2. Lack of correlation between mean ferritin and hs-CRP serum levels, at each diagnostic and monitoring stage, shows that they are independent and cannot be determined interchangeably.

## Figures and Tables

**Table 1:** The basic descriptive parameters and the results of the Shapiro-Wilk test and the *t*-test for the expected value.

Group	Parameter	Stage of the study	Estimators for descriptive parameters				S-W test	*t* test	
			$\overset{-}{x}$	SD	*x*_min⁡_	*x*_max⁡_	*P*	Norm	*P*
Group I treated anti-inflammatory two weeks after surgery	Ferritin (ng/mL)	Before surgery	75.4	33.8	30.5	132.1	>0.05	≤140 ≥45	**<0.001** **<0.001**
		2 weeks after surgery	64.7	30.3	21.5	120.9	>0.05	≤140 ≥45	**<0.001** **<0.01**
		6 weeks after surgery	52.4	21.3	21.7	91.0	>0.05	≤140 ≥45	**<0.001** >0.05
	hs-CRP (mg/L)	Before surgery	1.04	0.57	0.29	2.03	>0.05	≤3.0	**<0.001**
		2 weeks after surgery	0.88	0.49	0.15	1.84	>0.05	≤3.0	**<0.001**
		6 weeks after surgery	0.65	0.34	0.19	1.40	>0.05	≤3.0	**<0.001**

Group II patients treated only surgically, without anti-inflammatory therapy	Ferritin (ng/mL)	Before surgery	70.4	40.7	15.6	143.5	>0.05	≤140 ≥45	**<0.001** **<0.01**
		2 weeks after surgery	58.9	28.9	20.8	124.8	>0.05	≤140 ≥45	**<0.001** **<0.05**
		6 weeks after surgery	65.9	27.2	23.6	136.0	>0.05	≤140 ≥45	**<0.001** **<0.01**
	hs-CRP (mg/L)	Before surgery	1.28	0.52	0.37	2.60	>0.05	≤3.0	**<0.001**
		2 weeks after surgery	1.14	0.40	0.39	1.88	>0.05	≤3.0	**<0.001**
		6 weeks after surgery	0.92	0.48	0.23	1.98	>0.05	≤3.0	**<0.001**

**Table 2:** The mean values and standard deviations of ferritin concentrations in women, men and total patients before and 2 weeks after surgery.

Observation phase	Female	Male	F + M
Before surgery			
Mean	75.0	75.6	75.4
SD	46.3	31.7	33.8
2 weeks after surgery			
Mean	69.6	63.3	64.7
SD	48.8	25.7	30.3

**Table 3:** Statistical significance of Pearson's correlation between ferritin and hs-CRP concentrations.

Stage of follow-up	Correlation coefficient *r* *P* value	Group		
		I	II	Total
Before surgery	*r*	−0.154	0.355	0.092
	*P*	*P* > 0.05	*P* > 0.05	*P* > 0.05

2 weeks after surgery	*r*	0.386	−0.059	0.152
	*P*	*P* > 0.05	*P* > 0.05	*P* > 0.05

6 weeks after surgery	*r*	0.317	0.169	0.266
	*P*	*P* > 0.05	*P* > 0.05	*P* > 0.05
